# Is Serum Progranulin Level a Biomarker in Autism and Cognitive Development Disorders?

**DOI:** 10.5152/eurasianjmed.2022.21292

**Published:** 2022-02-01

**Authors:** Fatma Betül Özgeriş, Nezahat Kurt, Ilknur Ibili Ucuz, Kübra Koçak Yilmaz, Mevlüt Sait Keleş, Atilla Çayir, Onur Burak Dursun

**Affiliations:** 1 Department of Nutrition and Dietetics, Ataturk University School of Health Sciences, Turkey; 2 Department of Medical Biochemistry, Erzincan Binali Yildirim University School of Medicine, Turkey; 3 Department of Child and Adolescent Psychiatry, Inonu University School of Medicine, Malatya, Turkey; 4 Independent Researcher.; 5 Department of Medical Biochemistry, Uskudar University School of Medicine, İstanbul Turkey; 6 Department of Pediatric Endocrinology, Erzurum Regional Training and Research Hospital, Erzurum, Turkey; 7 Autism, Mental Special Needs and Rare Disease Department in the Turkish Ministry of Health, General Directorate of Health Services, Ankara, Turkey

**Keywords:** Autism spectrum disorders, cognitive developmental delay, progranulin

## Abstract

**Objective:** Cognitive developmental delay is a picture of the group of early-onset chronic diseases that affect 1.5-10% of children. Autism spectrum disorders are neurodevelopmental diseases with a genetic basis and abnormal brain development, characterized by disorders in areas that make up interpersonal relationships, such as communication, social cognition, and processing of emotional signals. Immune system dysfunction is thought to play a role in the pathogenesis of some neurological disorders, including autism. Progranulin is thought to be a regulator of the innate immune response. The purpose of this study was to look at plasma levels of progranulin, an anti-inflammatory neurotrophic factor, in children with autism spectrum disorder and cognitive developmental delay.

**Materials and Methods:** The study was conducted on 52 children who were patients and 35 healthy children. Of the 52 children of the patient group, 32 were diagnosed with CDD and 20 were diagnosed with cognitive developmental delay–autism spectrum disorder. Serum progranulin concentrations were measured using a human-specific sandwich enzyme-linked immunosorbent assay.

**Results:** Serum progranulin concentration was statistically lower in the patient group (110.746 ± 26.04) than in the healthy control group (137.346 ± 30.02). There was a statistically significant difference between the groups in levels of serum progranulin (*P* = .000). Receiver operating characteristic analysis was performed to evaluate the potential of progranulin as a biomarker to distinguish patients with cognitive developmental delay–autism spectrum disorder from healthy children. It detected a moderate area under the curve (0.743 ± 0.06) value and a more significant *P* value for progranulin (*P* = .000).

**Conclusion:** Progranulin deficiency in patients with autism spectrum disorder–cognitive developmental delay may result in decreased neurotrophic support for many years, with cumulative damage associated with unregulated inflammation that may play a role in autism spectrum disorder–cognitive developmental delay. We believe that low progranulin levels could be a biomarker for autism spectrum disorder–cognitive developmental delay.

**Main Points**

- This study evaluated the plasma levels of progranulin, an anti-inflammatory neurotrophic factor, in children with autism spectrum disorder (ASD) and cognitive developmental delay (CDD).
- Plasma progranulin levels were decreased in the patient group with ASD and CDD.
- Decreased progranulin levels suggest that it may be a biomarker for ASD–CDD.

## Introduction

Cognitive developmental delay (CDD) is a picture of the group of early-onset chronic diseases that affect 1.5-10% of children. Cognitive developmental delay is a subclass of developmental disorders characterized by a significant delay in 2 or more domains, including fine/gross motor domain, speech/language domain, cognition, social/personal domain, and activities of daily living.^1^

Autism spectrum disorders (ASD) are neurodevelopmental diseases with a genetic basis and abnormal brain development, characterized by disorders in areas that make up interpersonal relationships, such as communication, social cognition, and processing of emotional signals.^2^

Some neurological illnesses, such as autism, are thought to be caused by an immune system malfunction.^3,4^ Autoimmunity to the central nervous system may be a significant factor in ASD. This can be clearly inferred from the presence of brain-specific autoantibodies in some ASD children.^5,6^

Progranulin (PGRN), a glycoprotein-based growth factor, is involved in a variety of physiological and pathological processes, including growth and development,^7^ metabolic regulation, and wound healing.^8^ Recent studies have shown that PGRN may function as a neurotrophic factor involved in normal neuron biology.^9^ In the literature, PGRN has been hypothesized as a regulator of the innate immune response, but the variables that affect PGRN activity are yet unknown.^10^

Neutrophils, the body’s first line of defense, respond fast to tissue damage and invading germs by producing vast amounts of reactive oxygen species and releasing granular contents that kill infections.^11^ Progranulin is highly expressed on neutrophils and is converted into granulin peptides by neutrophil-released elastase.^12^ After cleavage, granulin peptides stimulate interleukin (IL)-8 expression in epithelial cells to recruit additional neutrophils to the site of inflammation. secretory leukocyte protease inhibitor (SLPI) binds with PGRN and inhibits the conversion of PGRN to granulin peptides by elastase, providing a switch to control innate immunity and inflammation.^13^

The goal of this study was to investigate the PGRN, an anti-inflammatory neurotrophic factor, levels in the blood of children with ASD and CDD.

## Materials and Methods

The approval for this research was obtained from the Clinical Research Ethics Committee of Atatürk University, Faculty of Medicine (B.30.2.ATA0.01.00/28). All study participants were given the opportunity to give their informed consent.

The study sample group consists of 52 children who were patients (of the 52 children, 32 are diagnosed with CDD and 20 are diagnosed with CDD–ASD) aged 2-5 years and 35 children without any health problems who applied to Atatürk University Faculty of Medicine, pediatrics, and child-adolescent mental health and diseases polyclinics with complaints of developmental delay.

All cases presenting with developmental delay were evaluated with detailed developmental histories, together with detailed psychiatric, pediatric, and neurological examinations and tests. Children with any organic pathology that would cause developmental delay were not included in the study.

Cases meeting the study inclusion criteria were evaluated by specialist child and adolescent psychiatrists based on the Manual of Mental Disorders Identification and Classification Diagnostic and Statistical Manual of Mental Disorders (DMS-5). The Denver II developmental test and the Ankara Developmental Test Inventory were applied to the cases. In addition, the families of the subjects were asked to fill in the Autism Behavior Checklist and the Behavior Evaluation Scale for children 1.5-5 years old (Child Behavior Checklist For Ages (CBCL) 1.5-5). As a result of these evaluations, 2 main diagnostic classes were established as those with only cognitive developmental delay and those with the coexistence of cognitive developmental delay and autism spectrum disorder (CDD–ASD).

Blood samples taken from patients and healthy children were put into gel-containing vacutainers and centrifuged at 3500 rpm for 10 minutes, and serum samples were stored at −80˚C until the day of analysis. Serum PGNR concentrations were measured using a human-specific sandwich enzyme-linked immunosorbent assay (Hu PGNR ELISA kit; Cat. No. MBS163502, MyBioSource, Belgium, San Diego, USA). Progranulin analysis was performed by multiplate reader spectrophotometer (City for XS Powerwave, BioTEK Hampton, USA).

### Statistical Analysis

Statistical analysis was performed using the Statistical Package for Social Sciences for Windows Version 20.0 (IBM Inc.; Chicago, Ill, USA). The variables were investigated using Kolmogrov–Simirnov test to determine the normal distribution. Descriptive analyses were presented using means and standard deviations (mean ± SD). *P* < .05 was considered to be statistically significant.

Receiver operating characteristic (ROC) analysis test was applied to determine whether the continuous variable could be used in the diagnosis and to determine the cut-off value, positive predictive value (PPV), negative predictive value, and area under the curve (AUC) value. The statistical significance level for all data was taken as *P* < .05.

## Results

Table 1 shows the demographic characteristics of the patient group and the healthy control group. There was no statistical difference between the mean age of the total patient group and the control group (*P* = .997). Of the 87 participants, 37.9% were female and 62.01% were male.

**Figure 1.**
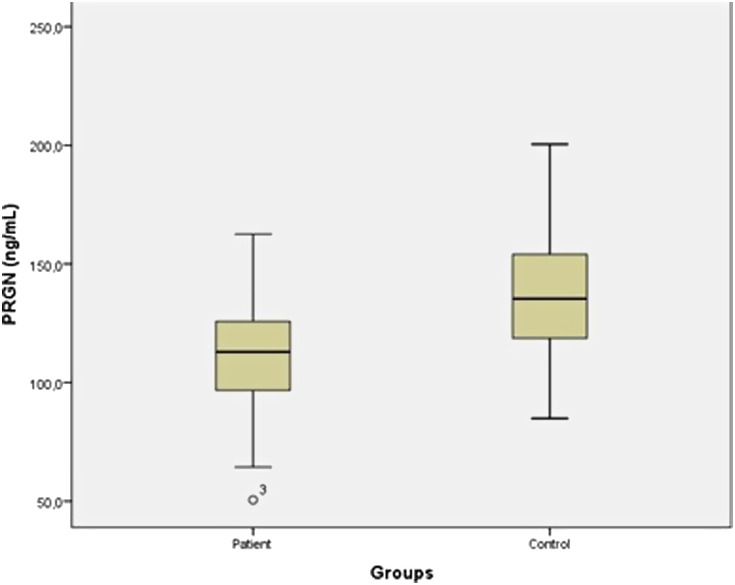
Serum progranulin levels of the control group and patient group.

Serum PGRN concentration was statistically lower in the patient group than in the healthy control group (*P* = .000). Box plot graphs of the patient group and healthy control group are given in Figure 1. There was no statistical difference between PGRN levels in patient subgroups (*P* = .563). Serum PGRN levels of all groups are presented in Table 2.

According to the findings, there was no statistically significant difference in serum PGRN levels between males and females in the patient and healthy groups (*P* > .05) (Table 3).

**Figure 2.**
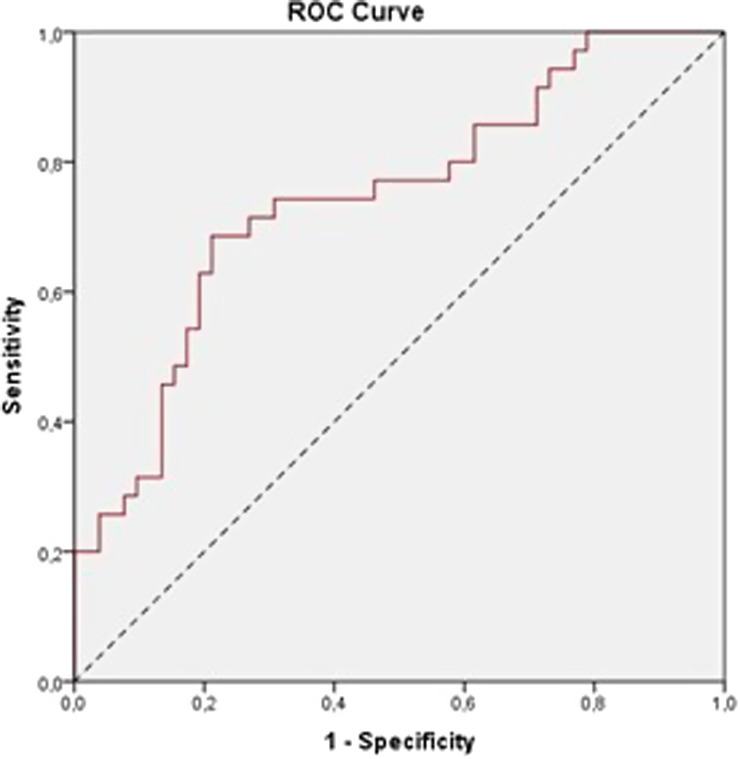
Receiver operating characteristic analysis graphic of progranulin.

Receiver operating characteristic analysis was performed to evaluate the potential of PGRN as a biomarker to distinguish CDD–ASD patients from healthy children. It detected a moderate AUC value and a more significant *P* value for PGRN (*P* = .000). The cut-off value was 155.334 ng/mL for PGRN, and PPV and PNV were calculated. The values of ROC analysis are presented in Table 4 and Figure 2.

## Discussion

The development of the brain includes a set of fundamental biological processes that occur in concert and are tightly regulated by area and time. Some genetic and/or environmental variables may interfere with these processes, causing variations from the expected path. Large-scale brain abnormalities are linked to both morphological and functional organization in ASD, according to previous clinical findings.^14,15^ Autism spectrum disorder and CDD may partially involve an autoimmune pathogenesis.^3^ It has a pathological role in neutrophillear autoimmunity.^16^ Progranulin, a neurotrophic factor expressed primarily in neurons and microglia, suppresses neutrophil activation and inflammatory activity, making it a significant anti-inflammatory suppressor.^17^

In our study, serum PGRN levels were found to be significantly lower in children with CDD-ASD compared to healthy controls (*P* = .000).

Because it has been shown that PGRN plays a vital role in the beginning and progression of neurodegenerative disorders, significant attention has been dedicated to the functional role of PGRN in the central nervous system in recent years.^18-21^ Progranulin is expressed broadly throughout early brain development, although it is eventually confined to certain neuronal populations such as cortical and hippocampal pyramidal neurons and Purkinje cells.^22^ The finding of PGRN gene null mutations as a prevalent cause of autosomal dominant tau-negative frontotemporal lobe dementia (FTLD) has piqued interest in the gene.^23^ As a result, neurodegeneration is hypothesized to be caused by haploinsufficiency with diminished PGRN-induced neuronal survival.^24^

According to new study, FTLD is caused in part by brain damage produced by a combination of dysregulated inflammation and increased neuronal sensitivity due to low PGRN levels.^25^ According to a research, PGRN/GRN is a neurotrophic factor that promotes neuronal survival and axonal development and that relative PGRN shortage in individuals with PGRN mutations alters neurite integrity and may result in neurodegeneration.^21^

Recent research focus on ASD has demonstrated that neuroinflammation is the underlying cause, with evidence from the dysregulated cytokine profiles in the cerebrospinal fluid of children with ASD.^26^ Neuroglial activation and an increase in inflammatory marker levels in the cerebrospinal fluid were found in postmortem brain samples from patients with ASD, although little is known about the underlying molecular mechanisms.^27^ Cell activity of PGRN is regulated by interaction with proteins of the cell membrane and extracellular matrix. Proteolytic enzymes play a crucial role in PGRN activity regulation. The neutrophil enzymes elastase and proteinase-3 are crucial in balancing the anti-inflammatory and inflammatory actions of intact PGRN and its peptide fragments containing granulin domains.^10^

Progranulin is vital for increasing long-term neuronal survival and brain availability as an effective neuroinflammatory regulator and autocrine neurotrophic factor domains of granülin. Although the mechanisms connecting PGRN deficit to ASD neurodegeneration are unknown, it is apparent that PGRN deficiency contributes to neurodegeneration, manifesting at an early stage in neurodegenerative illnesses and remaining stable over time. These properties suggest that PGRN might be a key therapeutic target, and restoring PGRN levels could be a good strategy to prevent and cure ASD.^28-30^

The etiology of ASD, a category of early-onset neurodevelopmental illnesses, is largely unknown. Autism is increasingly being recognized as a complex disorder caused by both hereditary and environmental factors. Autism is now diagnosed purely via clinical observation of changing behavior and can only be done around the age of 2 because clinical diagnosis in younger children is difficult and ambiguous. Therefore, there is a need for valid biomarkers that will allow us to improve and predict the diagnosis. Receiver operating characteristic analysis was performed to evaluate the potential of PGRN as a biomarker for CDD and ASD patients. It detected a moderate AUC (0.743) value and a more significant *P* value for PGRN (*P* = .000).

Thus, PGRN deficiency may result in diminished neurotrophic support for many years in certain people with ASD-CDD, with cumulative damage linked with dysregulated inflammation, which may play a role in autism.

Plasma PGRN levels were decreased in the patient group with ASD and CDD. In some patients with ASD-CDD, PGRN deficiency may result in decreased neurotrophic support for many years, with cumulative damage associated with unregulated inflammation that may play a role in ASD-CDD. To date, the diagnosis of ASD and CDD is based solely on clinical observation of changing behavior and can only be made around the age of 2 as clinical diagnosis in younger children is difficult and uncertain. There is a demand for reliable biomarkers that can help enhance and forecast diagnosis; furthermore, excellent biomarkers can predict the clinical outcome of ASD-CDD and aid in monitoring the efficacy of pharmacological and nutraceutical therapy. In conclusion, decreased PGRN levels suggest that it may be a biomarker for ASD and CDD.

### Limitation of the Study

The only limitation of this research is that the number of cases (especially ASD subgroups) is low.

## Figures and Tables

**Table 1.** Demographic Characteristics of the Control and Patients Groups

	PG			HCG
Variables	CDD (n = 32)	ASD-CDD (n = 20)	Total PG (n = 52)	(n = 35)
Age (months)	41.1 ± 1.06	42.1 ± 1.21	42.5 ± 1.16	46.6 ± 2.04
Gender (female/male)	14/18	4/16	18/34	15/20

PG, patient group; CDD, cognitive developmental delay; ASD-CDD, autism spectrum disorder-cognitive developmental delay; HCG, healthy control group.

**Table 2.** Progranulin Levels of the Healthy Control and Patients Groups

	PG				HCG	
Variables	CDD (n = 32)	ASD-CDD (n = 20)	*P*1 value	Total PG (n = 52)	HCG (n = 35)	*P*2 value
PRGN (ng/mL)	109.071 ± 25.56	113.426 ± 27.24	.563	110.746 ± 26.04	137.346 ± 30.02	**.000**

PG, patient group; CDD, cognitive developmental delay; ASD-CDD: autism spectrum disorder-cognitive developmental delay; HCG, healthy control group; *P*1, significance of the comparison of CDD and ASD in the PG group; *P*2, significance of the comparison of PG and HCG groups.

**Table 3.** Serum Progranulin Levels in Male and Female Patients and Healthy Group

Variables	PG		*P*1	HCG		*P*2
Gender	Male	Female		Male	Female	
PRGN (ng/mL)	110.39±28.62	111.40±21.05	.895	132.22 ± 29.21	144.18 ± 30.71	.249

PG, patient group; CDD, cognitive developmental delay; ASD-CDD, autism spectrum disorder-cognitive developmental delay; HCG, healthy control group; *P*1, significance of the comparison of female and male in the PG group; *P*2, significance of the comparison of female and male in the HCG group.

**Table 4.** Data of ROC Analysis for Progranulin

	AUC (CI%)	*P*	PPV (CI%)	NPV (CI%)
PRG	0.743 (87.6-98.6)	0.000	96.57 (98.3-87.4)	92.74 (94.5-88.7)

AUC, area under the curve; PPV, positive predictive value; NPV, negative predictive value.
